# UBXN1 interacts with the S1 protein of transmissible gastroenteritis coronavirus and plays a role in viral replication

**DOI:** 10.1186/s13567-019-0648-9

**Published:** 2019-04-27

**Authors:** Peng Yuan, Shilei Huang, Zhou Yang, Luyi Xie, Kai Wang, Yang Yang, Lin Ran, Qiuhan Yu, Zhenhui Song

**Affiliations:** Department of Veterinary Medicine, College of Animal Science, Southwest University, Chongqing, 402460 China

## Abstract

Transmissible gastroenteritis coronavirus (TGEV) is an enteropathogenic coronavirus that causes diarrhea in pigs and is associated with high morbidity and mortality in sucking piglets. S1 is one of two protein domains in the spike (S) glycoprotein and is responsible for enteric tropism, sialic acid recognition, and host receptor binding. Although there has been extensive research on the S1 protein of TGEV, little is known about the intracellular role of TGEV-S1. In the present study, we used yeast two-hybrid screening of a cDNA library from porcine intestinal cells to identify proteins that interact with TGEV-S1. Among 120 positive clones from the library, 12 intracellular proteins were identified after sequencing and a BLAST search. These intracellular proteins are involved in protein synthesis and degradation, biological signal transduction, and negative control of signaling pathways. Using a glutathione-*S*-transferase (GST) pulldown assay and Co-IP, we found that UBXN1 interacts with the S1 protein. Here, we observed that TGEV infection led to increased UBXN1 expression levels during the late phase of infection in IPEC-J2 cells. Inhibition of UBXN1 in IPEC-J2 cells via siRNA interference significantly decreased the viral titer and downregulated the expression of S1. UBXN1 overexpression significantly increased the viral copy number. Additionally, we provided data suggesting that UBXN1 negatively regulates IFN-β expression after TGEV infection. Finally, our research indicated that UBXN1 plays a vital role in the process of TGEV infection, making it a candidate target for the development of a novel antiviral method.

## Introduction

Transmissible gastroenteritis virus (TGEV) is a pathogenic agent of porcine transmissible gastroenteritis (TGE), which causes vomiting, diarrhea, and high mortality in suckling piglets, resulting in heavy losses to the pig breeding industry [1]. TGEV is an enteropathogenic coronavirus (order *Nidovirales*, family *Coronaviridae*) with a large, positive-sense, single-stranded RNA genome. The genome contains nine open reading frames (ORFs) encoding 16 kinds of nonstructural proteins and four structural proteins, spike (S), envelope (E), membrane (M), and nucleoprotein (N), which are arranged in the order of 5′-replicase-(1a/1b)-S-3a-3b-E-M-N-7-3′ [2]. The spike protein is the largest of the four structural proteins; as the main envelope glycoprotein on the virion surface [3], S plays a key role in the early infection of TGEV [4].

The S protein contains an N-terminal globular S1 domain and a C-terminal S2 region in the form of a stalk that connects S1 with the viral envelope [5]. In the early stage of TGEV infection, TGEV-S1 is mainly responsible for binding to cellular receptors, and the S2 domain is associated with membrane fusion between the virus and host cells. Compared with the S2 domain, the S1 domain has many significant functions similar to those of the entire S protein, including enteric tropism [6], sialic acid-binding activity [7], neutralizing antibody induction, and host receptor binding. Determinants for enteric tropism and sialic acid recognition are located in a 224 aa region at the N terminus of the TGEV S1 domain. In addition, the receptor binding domain (RBD) of TGEV, which resides in a 150 aa fragment at the C-terminus of the S1 region, recognizes and binds to the cellular receptor, porcine aminopeptidase N (pAPN). The four main antigenic sites (A to D) on the S protein are mostly contained within the S1 domain [8]. Interestingly, the immunogenicity of TGEV S1 is stronger than that of the whole S protein and is the major inducer of TGEV-neutralizing antibodies [9].

The features of TGEV-S1 mentioned above were indicated that it would be important for TGEV invasion of host cells. Many studies have examined the roles of TGEV-S1 during viral invasion of host cells; however, little is known about the intracellular function of TGEV-S1. In the present study, using a yeast two-hybrid system, we found that TGEV-S1 interacts with multiple intracellular proteins, including UBX domain-containing protein 1 (UBXN1).

UBXN1 is a member of the UBX family that is believed to regulate many cellular activities. The UBX family is characterized by low homology with ubiquitin at the amino acid level but has the same 3-dimensional structure as ubiquitin [10]. UBXN1 has the same structure as NSFL1C, UBXD7, UBXD8, and FAF1. All of these proteins feature an N-terminal UBA domain and a C-terminal UBX domain [11]. The N-terminal UBA domain binds and functions as a cofactor for valosin-containing protein (VCP), also known as p97, which plays a central role in many ubiquitin-mediated pathways and in numerous biological activities, including cell cycle and protein damage [12]. The C-terminal UBX domain has a strong inhibitory effect on the RNA virus-induced type I interferon response [13]. In the present study, we further confirmed that UBXN1 is associated with TGEV-S1 expression and TGEV replication, and our findings provide insight into the role of UBXN1 in TGEV infection.

## Materials and methods

### Strains, cells, and virus

Y187 yeast cells harboring the IPEC-J2 cDNA library and Y2HGold yeast cells harboring the TGEV-S1 gene (pGBKT7-S1) were constructed by our group [14]; the Y2HGold and Y187 cells were purchased from Clontech, Japan. Porcine small intestinal epithelial cells (IPEC-J2) and the TGEV Miller strain (TGEV Miller) were stored in the laboratory.

### Plasmids and antibodies

The prokaryotic expression vectors pGEX-4T-1 and pET-28a were provided by Jian Wu from the Lanzhou Veterinary Research Institute, (LVRI, Gansu Province, China). The anti-C-Myc antibodies, anti-GST-tag antibodies, and anti-His-tag antibodies were purchased from Sangon Biotech, China. Rabbit polyclonal antibodies (pAbs) recognizing UBXN1 and horseradish peroxidase (HRP)-conjugated goat anti-rabbit IgG were purchased from Proteintech, USA. Polyclonal antibodies recognizing the TGEV S1, and M proteins were donated by Yu Bai from the Wenzhou College of Science and Agriculture. Small interfering RNA (siRNA) and the Plenti-CMV-GFP-2A-Puro-UBXN1 overexpression plasmid were constructed by Longda Bio, China.

### Yeast two-hybrid system

Y2HGold (pGBKT7-S1) yeast were activated, and the baseline expression level of the S1 protein was assessed by Western blotting.

Yeast cells harboring the cDNA library were diluted at 1/10, 1/100, 1/1000, and 1/10 000, and 100 µL was spread onto SD/−Leu plates. The number of colonies that grew on the plates was counted to determine the library capacity. Twenty-four clones were randomly selected to test the quality of the prey library using PCR. The yeast two-hybrid assay was performed according to the instructions of the Matchmaker Gold Yeast Two-Hybrid System. Y2HGold (pGBKT7-S1) and Y187 (IPEC-J2 cDNA) cells were mixed and incubated at 30 °C for 3–5 days. The culture mixture was plated on SD/100 µL Trp/−Leu/−Ade. All white colonies that grew were plated onto higher-stringency SD/−Trp/−Leu/−Ade/−His/X-α-Gal plates. Finally, all blue colonies that grew on SD/−Trp/−Leu/−Ade/−His/X-α-Gal/AbA plates were selected and inoculated into YPDA liquid medium. The positive prey plasmids were rescued, and their cDNA inserts were sequenced to identify the candidate proteins.

### GST pulldown assay

Under induction by 1 mM isopropyl-b-d-thiogalactopyranoside (IPTG), glutathione-*S*-transferase (GST)-S1 was expressed by *E. coli* strain Rosetta harboring pGEX-4T-S1, which was constructed using the indicated primers (Table 1). The porcine UBXN1 gene was amplified by PCR using the indicated specific primers (Table 1) and cloned into pET-28a to generate pET-28a-UBXN1, which expressed His-UBXN1. GST-S1 and His-UBXN1 were coincubated at 4 °C for 8 h. The mixture was then subjected to GST pulldown using GST spin columns. The eluted proteins were analyzed via 10% polyacrylamide gel electrophoresis (PAGE).

**Table 1:** **Primers used to construct the recombinant plasmids**

Gene	Accession number	Purpose	Sequence (5′–3′)
TGEV-S1	DQ811786.2	Construction of pGBKT7-S1	F: CTCTTCCAGCCCTCCTTCC
			R: GGTCCTTGCGGATGTCG
TGEV-S1-t	XM_003353824.1	Construction of pGEX-4T-S1	F: CGGAATTCGAGAAGGCTCTGGCCCTCA
			R: GGCGCATTTCGTCTTCCTGG
UBXN1-t	DQ811786.2	Construction of pET-28a-UBXN1	F: TCGGTCGACTCAACTGGGACA-CTTCTT
			R: GCGGTCGACTTAGTTTGTCTAATAATA

### Coimmunoprecipitation (Co-IP) assay

The lysate of IPEC-J2 cells infected with TGEV Miller for 36 h was prepared with RIPA lysis buffer (Proteintech) containing the protease inhibitor phenylmethanesulfonyl fluoride (PMSF) (1 mM). After centrifugation at 10 000 ×* g* for 20 min and measurement of the protein concentration using the BCA method, the lysate supernatant was pretreated with protein A/G PLUS-Agarose (Proteintech) for 60 min at 4 °C to purify the protein. The lysate supernatant (700 μg) was incubated with 3 μg of a rabbit pAb against UBXN1 overnight at 4 °C. Next, 10 μL of Protein A/G PLUS-Agarose was added to this mixture and incubated with shaking at 4 °C for 4 h. After washing four times with lysis buffer, the eluted proteins were analyzed by SDS-PAGE and Western blotting using pAbs recognizing the S1 protein of TGEV and rabbit pAbs recognizing UBXN1. The lysate of IPEC-J2 cells uninfected with TGEV was used as the control.

### Small interfering RNA (siRNA) assays

siRNA targeting *UBXN1*, 5′-CCTCATCGAGATGGGCTTT-3′ (produced by RIBO BIO, China), was transfected into susceptible IPEC-J2 cells, which were cultured overnight in six-well plates. Transfection with siRNA was performed using Lipofectamine™ 3000 (Thermo Fisher, USA) according to the manufacturer’s instructions. The transfection complex (8 nM siRNA) was added and incubated for 24 h, followed by removal of the culture supernatant. The collected samples were analyzed by Western blotting using a rabbit pAb recognizing UBXN1 as the primary antibody and HRP-conjugated goat anti-rabbit IgG as the secondary antibody. The siRNA sequence that caused the largest reduction in the expression of UBXN1 was used in subsequent studies of TGEV infection.

### Overexpression of UBXN1

IPEC-J2 cells were cultured overnight in six-well plates at 37 °C, and the prepared plasmid Plenti-CMV-GFP-2A-Puro-UBXN1 (2.67 μg) was then transfected into the cells for 36 h. Then, the cells were incubated with TGEV Miller (MOI, 0.1) at 37 °C and 5% CO_2_. The cells were collected after 24 h, and the lysate of IPEC-J2 cells was analyzed using absolute quantitative PCR to determine the TGEV copy number, as described above.

### TCID_50_ assay

Transmissible gastroenteritis virus-infected cells were collected after treatment with the interference fragment and overexpression plasmid for 24 h. The viral titers and TGEV copy numbers were determined at different time points, and the growth kinetics curves were plotted. The cells were subjected to three freeze–thaw cycles, diluting 7 concentrations by tenfold gradient from 10^−1^ to 10^−7^, and added to 96-well plates. Each dilution was added to eight replicate wells. The viral titer was measured in the TGEV-infected, virus-infected-interference and virus-infected-overexpression groups. The method of Reed and Muench was then used to calculate the TCID_50_ of the virus for the different groups.

### Real-time quantitative PCR

At 12 h after incubation with TGEV at a multiplicity of infection (MOI) of 0.1 in each well of 6-well plates, total RNA was extracted from the cells using RNAiso Plus reagent (Invitrogen, USA) and subsequently reverse transcribed to cDNA (4 ng) using PrimerScript™ RT Master Mix (Takara, Japan). The β-actin and UBXN1 genes were subjected to quantitative real-time PCR using gene-specific primers (Table 2). The cycling conditions were as follows: 95 °C for 30 s, followed by 40 cycles at 95 °C for 5 s and 55 °C for 30 s. A negative control was included in each run, and the specificity of the amplification reaction was tested by melting curve (Tm value) analysis. The relative expression levels of the three genes were compared using the 2^−∆∆^Ct method [15].

**Table 2:** **Primer sequences for real-time quantitative PCR**

Gene	Accession number	Sequence (5′–3′)	Size (bp)	Concentration
β-Actin	XM_003124280.2	F: CTCTTCCAGCCCTCCTTCC	97	0.5 µM
		R: GGTCCTTGCGGATGTCG		
UBXN1	XM_003353824.1	F: TTGGAGCTTGTGGCCCAGAA	139	
		R: GGCGCATTTCGTCTTCCTGG		
TGEV-N	NC_038861.1	F: TTCAACCCCATAACCCTCCAACAA	136	
		R: GGCCCTTCACCATGCGATAGC		

### Absolute quantification of TGEV by real-time PCR

To detect viral replication, we constructed a recombinant plasmid containing the N gene and used the TGEV-N gene to establish a relationship between the copy number (X) and the Ct (Y) value. Total viral RNA was prepared from the collected cells as described above. The primers for TGEV-N (Table 2) were used to quantify the TGEV copy number via absolute quantitative PCR conducted with Power SYBR Green PCR Master Mix (Takara, Dalian, China) according to the manufacturer’s instructions. All data were obtained with a mini Q-PCR machine and analyzed with GraphPad Prism 6 software based on the cycle threshold (^∆∆^Ct) method.

### Western blotting

siRNA targeting *UBXN1* was transfected into IPEC-J2 cells in six-well plates. After 24 h, the cell culture was infected with TGEV Miller at a MOI of 0.1 and incubated for 24 h. Then, 200 μL of lysis buffer RSVP (containing 1 mM PMSF) was added into each well. The cells were scraped and collected into tubes, and the tubes were then incubated on ice for 30 min. The lysis products were centrifuged at 10 000 ×* g* and 4 °C for 10 min to prepare samples for Western blotting.

### Enzyme-linked immunosorbent assay (ELISA)

IPEC-J2 cells were seeded in six-well plates at a density of 1.0 × 10^5^ cells per well. When grown to a confluence of approximately 50–60%, the cells were separately transfected with either the overexpression plasmid or siRNA1. Cells with siRNA interference were incubated with TGEV Miller at an MOI of 0.1 for 24 h. Cells transfected with the overexpression plasmid were incubated with TGEV Miller for 36 h The lysate supernatant of IPEC-J2 cells was collected at 1 h, 12 h and 24 h after TGEV infection. NC was established as the blank control, and cells infected with TGEV were used as the infection control. Each experiment was performed in triplicate. The collected samples were centrifuged at 1000 × *g* for 20 min, and the supernatants were added to each well of a 96-well plate. The wells were coated with 100 µL of conjugate reagent at 37 °C for 60 min. Subsequently, each well was washed three times with wash buffer. Prior to adding stop solution, chromogenic agents A and B were sequentially added to each well. The assay was conducted according to the manufacturer’s instructions (MEIMIAN, China) and analyzed at a wavelength of 450 nm with correction at 570 nm.

### Statistical analysis

All results in the figures are presented as the means ± standard deviations (SDs) of three independent experiments, using GraphPad Prism (GraphPad Software 6, Inc.) For each assay, a t-test was used for statistical comparison, and a *p* value of < 0.05 was considered statistically significant.

## Results

### Identifying host proteins interacting with TGEV-S1 via a two-hybrid assay

**Figure 1:**
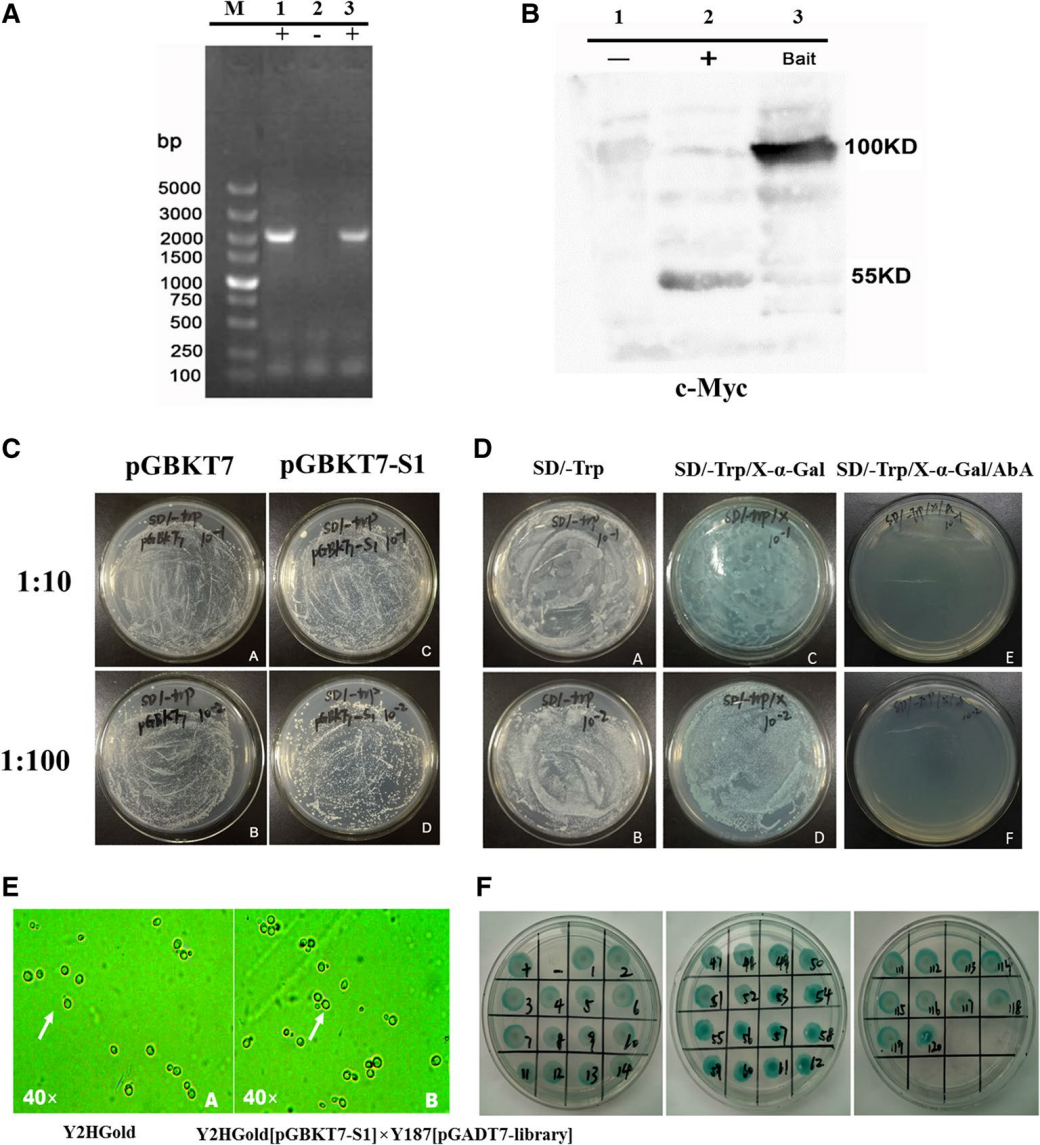
**Testing bait protein expression, autoactivation, and toxicity. A** Yeast colony identification by PCR. M: DNA marker DL5000; 1–2: PCR of colonies transformed with pGBKT7-S1; −: blank control. **B** Expression of S1 in yeast by Western blotting. 1: pGBKT7 negative control; 2: pGBKT7-53 positive control; 3: pGBKT7-S1 expressed protein. **C** Toxicity assessment of the pGBKT7-S1 bait plasmid. Yeast cells harboring pGBKT7-S1 and the pGBKT7 control plasmid were spread at two dilutions (1:10 and 1:100) on SD/Trp plates, and the size and number of the colonies were compared to evaluate the toxicity of the bait plasmid. **D** Identification of autoactivation of the pGBKT7-S1 bait plasmid. Yeast cells harboring pGBKT7-S1 were spread at 1/10 and 1/100 dilutions on SD/−Trp, SD/−Trp/X-α-Gal, and SD/−Trp/X-α-Gal/AbA plates for autoactivation testing. **E** A yeast two-hybrid system was used to screen for S1-interacting proteins. Diploids after yeast two-hybrid screening. The zygotes typically displayed a three-lobed structure similar to a “Mickey Mouse” face at 40× magnification. **F** The third screen was conducted using SD/−Trp/−Leu/−Ade/−His/X-α-Gal/AbA selective medium.

The bait vector pGBKT7 in the yeast two-hybrid system was constructed to screen the interactions between the TGEV-S1 protein and host proteins, and the recombinant plasmid was successfully transformed into Y2HGold yeast cells (Figure 1A). Western blotting showed that pGBKT7-S1 produced a fusion protein of approximately 100 kDa (Figure 1B). Yeast cells harboring pGBKT7-S1 and those harboring pGBKT7 (control) were spread at two dilutions onto SD/Trp plates, and the size and number of yeast colonies produced by pGBKT7-S1 were compared with those of colonies produced by the control pGBKT7. The results suggest that the bait vector was not toxicity to the yeast (Figure 1C). Yeast cells harboring pGBKT7-S1 were spread at two dilutions on plates of three different selective media. No colony growth was observed on the SD/−Trp/X-α-Gal/AbA plates, indicating that there was no self-activation phenomenon for the reporter genes (Figure 1D). These results indicate that the constructed pGBKT7-S1 bait vector could be used the yeast two-hybrid system for screening host proteins that interact with the TGEV-S1 protein. TGEV-S1 protein-associated cellular proteins were identified using the yeast two-hybrid system. The zygotes typically displayed a three-lobed structure similar to a “Mickey Mouse” face at 40× magnification (Figure 1E) after the bait strains Y2HGold (pGBKT7-S1) and Y187 (pIECs cDNA) were mixed and incubated. After three rounds of screening on plates with different nutrient-deficient media, a total of 120 blue clones that grew on the SD/−Trp/−Leu/−Ade/−His/X-α-Gal/AbA plates were visible (Figure 1F). Finally, twelve candidate proteins were available for a BLAST search of the sequencing results (Table 3).

**Table 3:** **BLAST search results for the positive clones**

Number of repeats	Protein name	NCBI accession no.
22	UBX domain-containing protein 1	XM_003353824.1
12	Kinesin-like protein KIF2C	XP_014384723.1
4	Calcium-binding protein 2-like	XP_019950966.1
2	DUF1410 domain-containing protein	WP_069096724.1
4	Bifunctional glutamate–proline—tRNA ligase	AAH88324.1
22	Asparaginyl-tRNA synthetase (NARS2)	AAH30041.1
8	Microsomal glutathione *S*-transferase 1	NP_999465.1
4	Glutaminyl-tRNA synthetase (GlnRS)	AAU85385.1
2	Tau-DTfdA family dioxygenase	WP_034307978.1
8	Bifunctional aminoacyl-tRNA synthetase	EGV92541.1
22	Asparagine—tRNA ligase	XP_021085155.1
2	Sensor histidine kinase	WP_018684459.1

Among these positive clones, the sequences of eight clones produced no BLAST results, and 12 different BLAST results were obtained from the remaining clones.

### UBXN1 interacts with the TGEV-S1 protein

**Figure 2:**
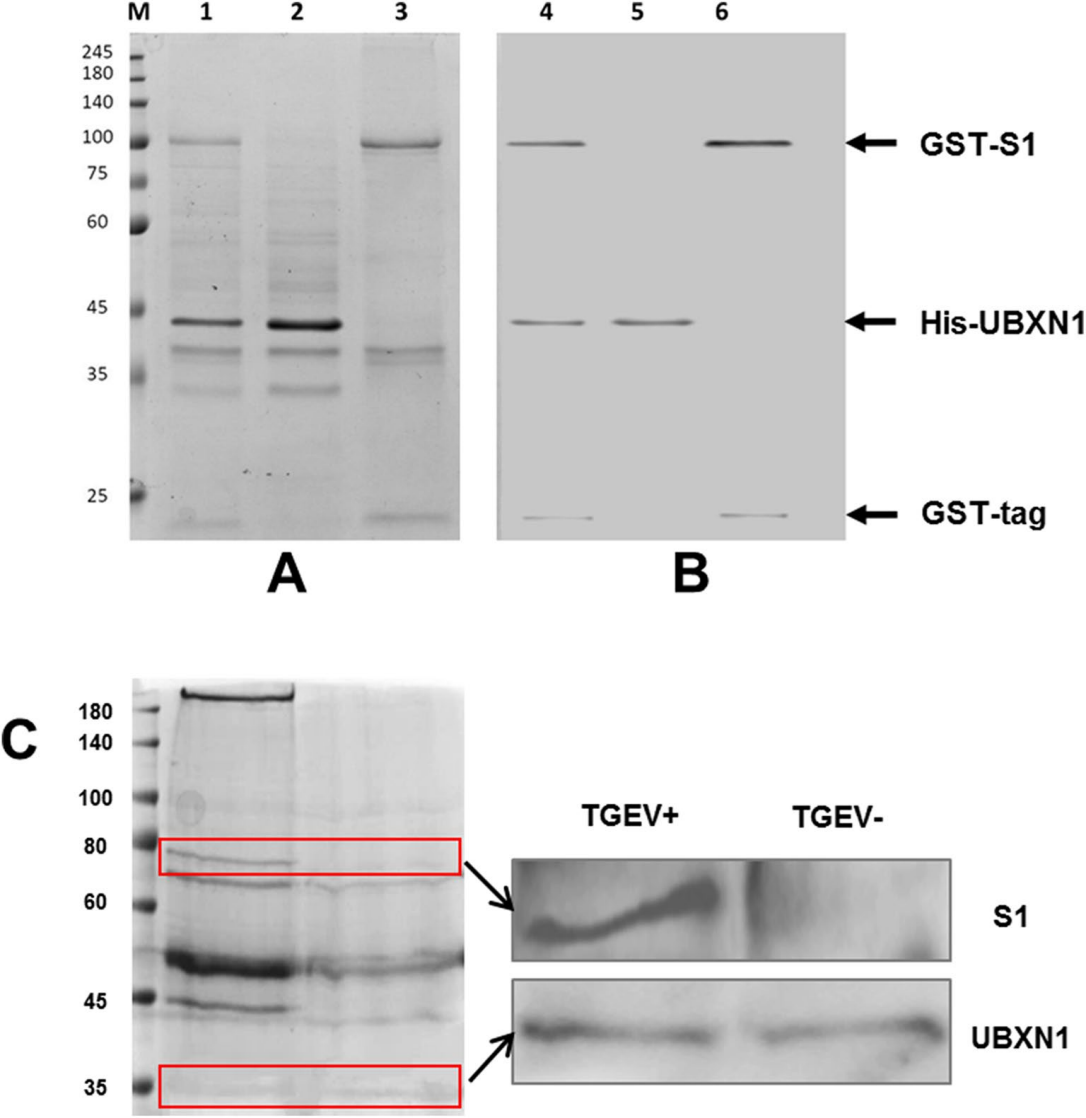
**SDS-PAGE (A) and Western blotting (B) results of the glutathione-*S*-transferase (GST) pulldown assay and CO-IP assay.** The GST-S1 and His-UBXN1 fusion proteins were used in the GST pulldown assay to verify the interaction between S1 and UBXN1. Lanes 1 and 4: result of S1 and UBXN1 pulldown; lanes 2 and 5: His-UBXN1 fusion protein (42 kDa); lanes 3 and 6: GST-S1 fusion protein (97 kDa) and GST tag blank control (20 kDa). **C** Co-IP assay for the interaction between S1 and UBXN1 in vivo. IPEC-J2 cells were infected with TGEV Miller for 36 h and were then subjected to a Co-IP assay. The coimmunoprecipitated proteins were detected by SDS-PAGE (left) and Western blotting (right). +: virus-infected group; −: normal group.

Next, the interaction between the S1 protein and candidate protein UBXN1 was verified using a GST pulldown assay and Co-IP. The His-UBXN1 fusion protein solution was added to Glutathione™4B agarose beads adsorbed with the GST-S1 fusion protein at the same concentration. The GST protein was used as the control to eliminate nonspecific protein binding. As shown in Figure 2A, the SDS-PAGE and Western blotting results revealed that the UBXN1 protein was present in the gel from the GST-S1 protein-immobilized beads but not in the other gels, and the Western blot indicated that the size of the His-UBXN1 fusion protein was approximately 42 kDa (Figure 2B). A Co-IP assay was performed to further verify the interaction between UBXN1 and TGEV-S1 in vitro. The Western blot (right) shown in Figure 2C indicates that the S1 protein was precipitated by the antibody in the TGEV-infected group but not in the mock-treated group. These results confirm the interaction between UBXN1 and the S1 protein both in vitro and in vivo.

### TGEV infection leads to increased UBXN1 expression levels

**Figure 3:**
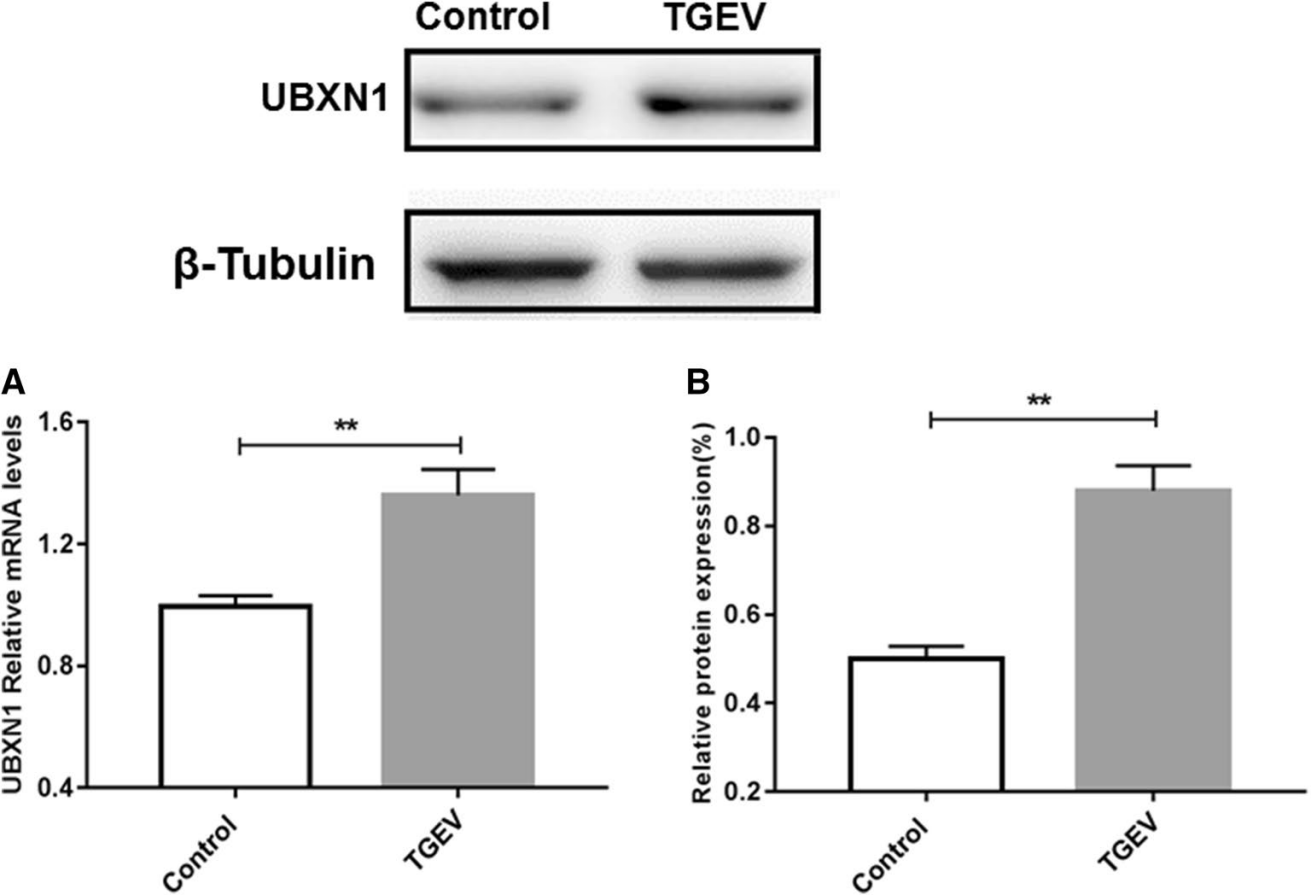
**TGEV infection led to increased UBXN1 expression levels.** Cell and protein samples from the group infected with TGEV for 24 h and the blank control group were collected, and the mRNA and protein levels of UBXN1 were determined. **A** Relative mRNA levels of UBXN1. **B** Relative protein expression of UBXN1. Control: blank group; TGEV: TGEV-infected group.

We confirmed that there was indeed an interaction between TGEV-S1 and UBXN1 by GST pulldown and Co-IP assays in vitro and in vivo. However, it was not clear whether this interaction was involved in the infection or replication of TGEV. Therefore, we infected IPEC-J2 cells with TGEV at an MOI of 0.1 and observed the changes in the UBXN1 mRNA and protein levels. The results showed that the UBXN1 mRNA and protein levels were significantly increased in IPEC-J2 cells after TGEV infection for 24 h (Figure 3).

### UBXN1-mediated infection and replication of TGEV

**Figure 4:**
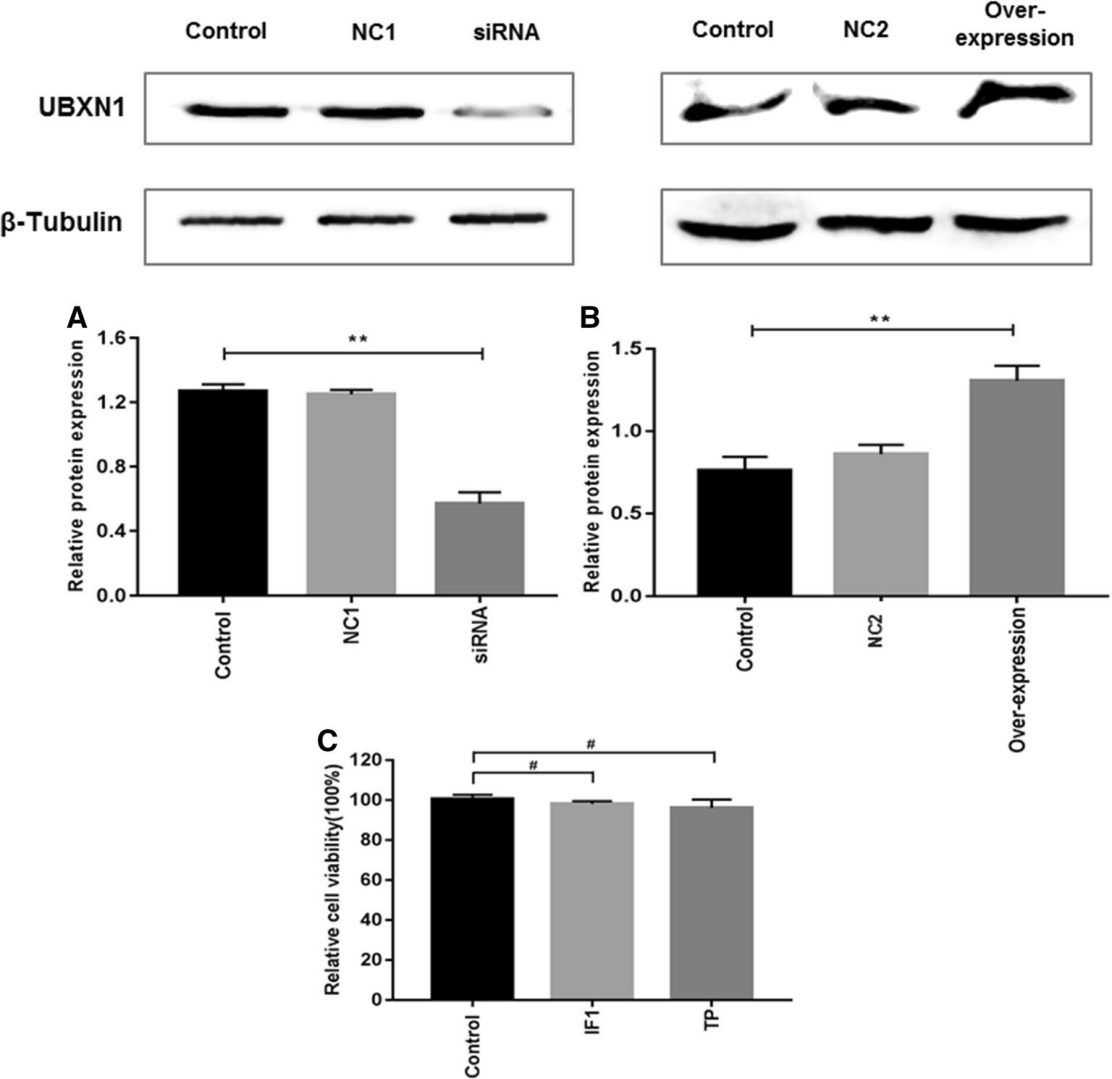
**Interference and overexpression of UBXN1. A**, **B** siRNA interference fragments reduced the expression of UBXN1. The relative expression of UBXN1 was assessed by Western blotting after 24 h of interference. Control: normal group; NC1: transfection reagent group; siRNA: fragment 1. **B** The lentiviral plasmid overexpressed UBXN1. The plasmid was transfected into cells for 36 h, and the relative expression of UBXN1 was assessed by Western blotting. Control: normal group; NC2: transfection reagent group; Overexpression: transfection plasmid group. **C** Molecular modulators of UBXN1 do not affect cell viability. Cell viability was determined by an MTT assay after treatment with the interference fragment (SiRNA) or overexpression plasmid (Plenti-CMV-GFP-2A-Puro-UBXN1) for 48 h. The data are the means ± SEMs of three independent experiments (t-test, ^#^*p* > 0.05). Control: normal group; IF1: interference fragment 1 group; TP: transfection plasmid group.

We determined that TGEV infection caused the upregulation of UBXN1 expression. We hypothesized that UBXN1 might mediate the infection and replication of TGEV, thus controlling the expression of UBXN1, which might cause corresponding changes in TGEV. The final result confirmed our hypothesis. We introduced interference and overexpression (Figures 4A and B) techniques to inhibit and overexpress UBXN1, and Figure 4C shows that the transfection reagents and overexpression reagents that we used were not toxic to the cells.

**Figure 5:**
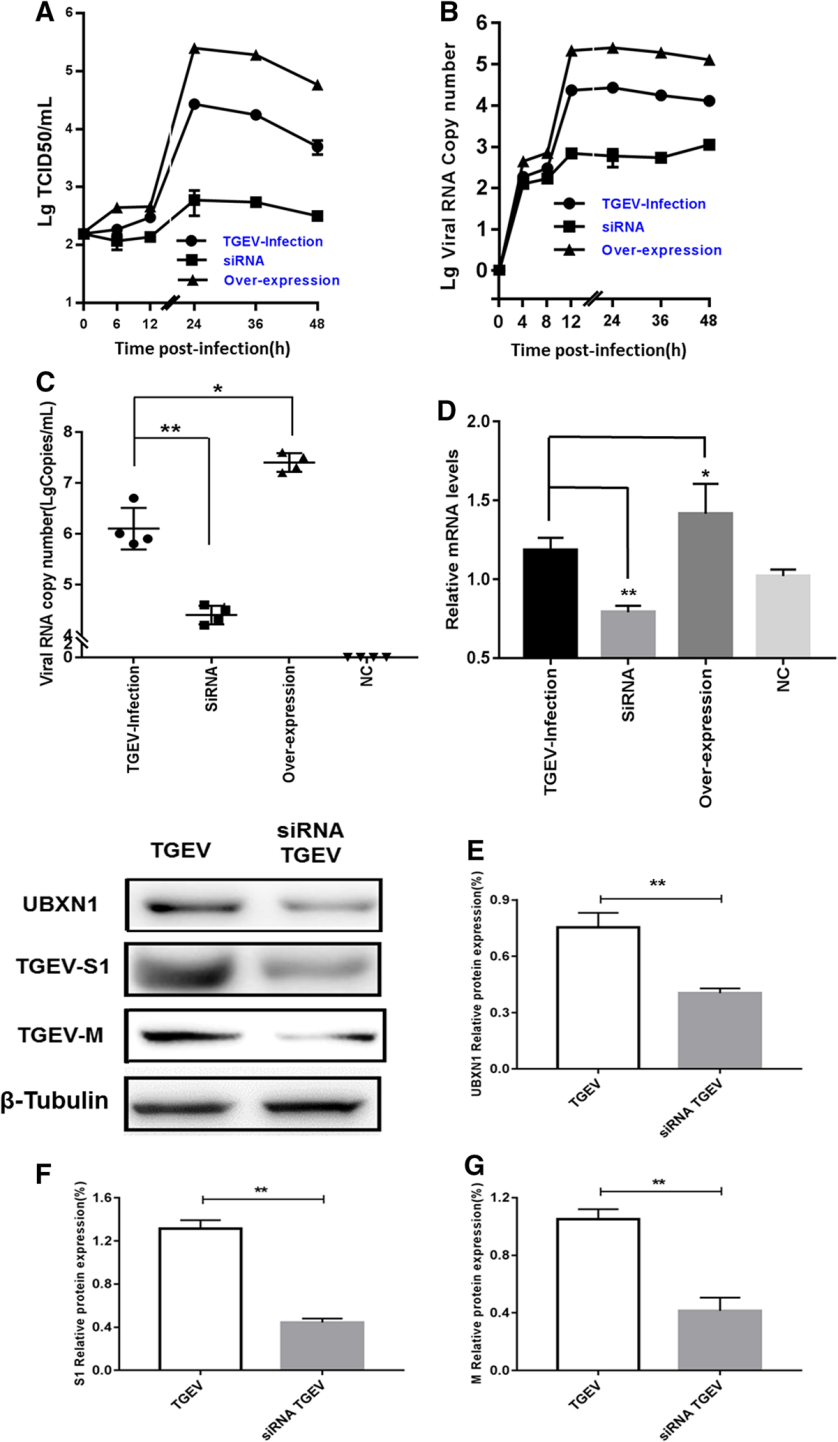
**UBXN1 mediated the infection and replication of TGEV. A**, **B** Generation of the viral proliferation curves in IPEC-J2 cells under different conditions. The cells were transfected with siRNA interference fragments and overexpression plasmids for 24 h and were then infected with virus to measure the TCID_50_ and copy numbers. **C**, **D** Quantitative real-time reverse transcription PCR results. At 12 h after the cells were incubated with TGEV Miller, qRT-PCR was performed to measure the mRNA expression levels of TGEV N, UBXN1 and β-actin. The standard curve based on N was used to examine the TGEV copy number. TGEV-infection: virus-infected group; siRNA: virus-infected siRNA interference group. Overexpression: virus-infected overexpression group. NC: blank group. **E**–**G** Western blotting results after siRNA interference and viral infection. At 24 h after siRNA interference, the cells were incubated with TGEV Miller. After 48 h, the cells were harvested and assessed by Western blotting for UBXN1, S1, and M protein expression. TGEV: virus-infected group; siRNA TGEV: virus-infected siRNA interference group.

We generated the viral growth curves of TGEV under normal, interference and overexpression conditions (Figures 5A and B). We first observed changes in the titer of TGEV. After siRNA interference and overexpression for 24 h, IPEC-J2 cells were infected with TGEV for 12 h, and the replication of TGEV was investigated by a TCID_50_ assay. The result shows that the TCID_50_ in the siRNA group (TCID_50_ = 10^2.9^/mL) was lower than that in the virus-infected group (TCID_50_ = 10^4.3^/mL). The TCID_50_ in the overexpression group (TCID_50_ = 10^5.4^/mL) was higher than that in the virus-infected group (TCID_50_ = 10^4.3^/mL) (Figure 5A).

To further verify our hypothesis, we then detected the levels of TGEV mRNA and protein under the conditions of UBXN1 gene silencing and overexpression.

The harvested cells were examined by absolute quantitative PCR. The equation for the standard curve based on the N gene as the target gene for measuring the viral copy number was y = − 3.602 × X + 40.5. The expression level of TGEV-N declined when the expression of UBXN1 was inhibited, and the viral copy number was 1.84 × 10^3^ copies/mL, lower than that in the TGEV-infected group; the TGEV copy number in the overexpression group was 7.32 × 10^5^ copies/mL, higher than that in the TGEV-infected group (Figure 5C). In addition, the mRNA level of UBXN1 also showed the same trend as the copy number of TGEV (Figure 5D).

Considering that the expression of UBXN1 was upregulated by TGEV infection, we mainly explored the changes in TGEV at the protein level by inhibiting UBXN1 expression via siRNA interference (Figure 5E). Protein samples were collected from the virus-infected group and the virus-infected siRNA interference group and examined by Western blotting. The results showed that the S1 and M protein levels in the virus-infected siRNA interference group were lower than those in the virus-infected group (Figures 5F and G).

The final results showed that UBXN1 interference and overexpression could decrease and increase the replication of TGEV, respectively. There was a positive correlation between UBXN1 expression and TGEV replication in IPEC-J2 cells. The viral titer, viral copy number and protein levels of S1 and M all showed to be associated with UBXN1 expression.

### UBXN1 negatively regulates IFN-β production induced by TGEV infection

**Figure 6:**
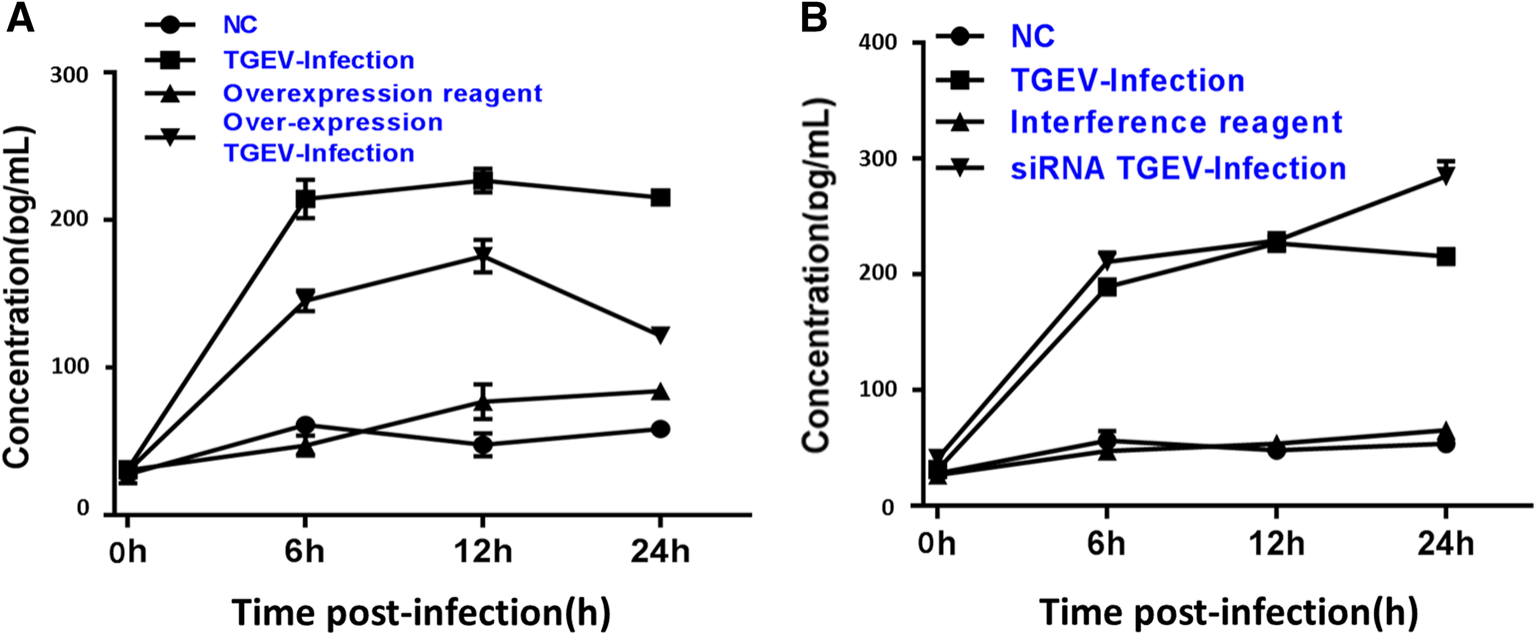
**IFN ELISA results. A** IFN production was measured at 0 h, 6 h, 12 h and 24 h after TGEV infection in cells overexpressing UBXN1. **B** IFN production was measured at 0 h, 6 h, 12 h and 24 h after TGEV infection in cells transfected with UBXN1 siRNA. NC: normal group; TGEV-infected: virus-infected group; siRNA: virus-infected siRNA interference group; Overexpression: virus-infected overexpression group; Overexpression reagent: group with UBXN1 overexpression without TGEV infection; Interfering reagent: group with treated with UBXN1 siRNA without TGEV infection.

It is well known that TGEV infection can induce cells to produce a large amount of interferons, and UBXN1 has a negative regulatory effect on the expression of type I interferon. Previously, we indicated that the expression of UBXN1 is upregulated by TGEV infection. Therefore, we hypothesized that TGEV may inhibit interferon expression by upregulating UBXN1 expression. We measured the expression of IFN-β after siRNA interference with and overexpression of UBXN1 without TGEV infection. The results confirmed that neither interfering with nor overexpressing UBXN1 caused a change in IFN-β expression without induction by TGEV. However, in the setting of TGEV infection, interference with and overexpression of UBXN1 greatly changed the expression of IFN-β. Overexpression of UBXN1 decreased IFN-β expression (Figure 6A), while interference with UBXN1 expression increased IFN-β expression (Figure 6B).

## Discussion

In the present study, most of the 20 proteins identified to interact with TGEV-S1 are involved in the synthesis and transport of proteins, biological signal transduction, and negative regulation of signal pathways. Five of the screened proteins were aminoacyl-tRNA synthetases (ARSs). ARSs are widely expressed among organisms and are responsible for catalyzing the attachment of amino acids to their cognate tRNAs [16], and the role of aminoacyl-tRNA synthetases in TGEV infection has already been explored [17]. Microsomal glutathione transferase 1 (MGST1) is a membrane-associated protein in the eicosanoid and glutathione metabolism (MAPEG) superfamily, which are glutathione-*S*-transferases (GSTs) [18]. MGST1 is widely distributed, with high levels in the endoplasmic reticulum (ER) and outer mitochondrial membrane (OMM). MGST1 is an ER-membrane-bound enzyme [19, 20]. MGST1 participates in a variety of physiological activities, including cellular defense against toxic, carcinogenic, and pharmacologically active electrophilic compounds (thereby protecting cells against H_2_O_2_-induced cell trauma) [2, 21]. The sensor histidine kinase is a membrane receptor with the highest diversity among all biological activities of microorganisms [22]. The receptor region of this protein is responsible for structural dynamics in control signal regulation [23]. This receptor functions in two-component signaling systems (TCSs); tens of thousands of TCSs are necessary for many aspects of biological activities in cells, including growth and survival [24]. Kinesin-like protein (KIF2C) belongs to the kinesin superfamily proteins (KIFs) and has the features of a highly conserved structural domain and a core that catalyzes hydrolysis. KIF2C plays a significant role in intracellular transport systems as a molecular motor that transmits the energy released from chemical reactions into mechanical motion [25]. Calcium-binding protein 2-like regulates its own biological activities via a reversible interaction of its specific protein-binding sites with Ca^2+^ [26]. Ca^2+^ is a common intracellular signal carrier that regulates many cellular processes from cell birth, differentiation, and reproduction to apoptosis [27].

In our screening results, UBXN1 appeared at a high frequency among the candidate proteins and was known to have a strong inhibitory effect on the RNA virus-induced type I interferon response [13]. Therefore, the candidate protein UBXN1 was selected as protein of interest for further verification of its interaction with TGEV-S1. Recently, UBXN1 was identified as a negative regulator in the tumor necrosis factor alpha (TNF-α)-mediated signaling pathway [11]. In the process of TGEV infection of IPEC-J2 host cells, the RNA of the single-stranded, positive-sense RNA virus TGEV is recognized as a viral pathogen-associated molecular pattern (PAMP). PAMPs are mainly regulated by cytoplasmic receptors, such as RIG-I-like receptors (RLRs) and melanoma differentiation-associated protein 5 (MDA5) [28]. Once the viral RNA is recognized, RIG-1 and MDA5 trigger a signal cascade reaction, stimulating the production of cytokines and chemokines. Type I interferons (IFNs) are quickly produced to establish the antiviral status in host cells. Mitochondrial antiviral-signaling protein (MAVS) (also known as IPS-1, CARDIF, and VISA) is an important protein in the RLR signaling pathway. MAVS binds to viruses via an interaction between the N-terminal caspase recruitment domain (CARD) of RLRs. Then, the TRAF functional module (TIM) recruits the E2 ligases TRAF3 and TRAF6, which activate canonical IκB kinases (IKKs) and IKK-related kinases such as TBK1 and IKKε, and eventually promoted the production of type I IFNs and proinflammatory factors [29].

Mitochondrial antiviral-signaling protein, as a central adaptor of RLRs, is negatively regulated by UBXN1. After infection with TGEV, UBXN1 is induced to recruit MAVS. UBXN1 blocks the binding site of TRAF3/6 (aa 450–480), which is important for MAVS-mediated regulation of the IFN induction. The binding of UBXN1 to MAVS interferes with intracellular MAVS oligomerization and blocks the formation of the MAVS/TRAF3/TRAF6 signalosome to inhibit the type I IFN response [2]. It has been confirmed that the N terminus of UBXN1 is responsible for binding to MAVS (aa 438–467) and that UBXN1’s functions in ubiquitination are independent of binding to VCP/p97 [30].

We confirmed the interaction between TGEV-S1 and UBXN1 using yeast two-hybrid screening, which suggested that TGEV-S1 might participate in UBXN1-related intracellular biological functions. UBXN1 directly bound to S1 in vitro and in vivo, as determined by the GST pulldown and Co-IP assays. Moreover, we found that TGEV-S1 and UBXN1 had a positive association. TGEV infection triggered an increase in the UBXN1 levels, and UBXN1 promoted TGEV proliferation, as proven by the siRNA and overexpression assays. We deduced that the binding of TGEV-S1 to UBXN1 induced UBXN1 expression to enhance TGEV proliferation. TGEV infection induces massive production of IFN-I in host cells, but it also results in a variety of immune escape mechanisms with IFN resistance. Finally, we confirmed by siRNA and overexpression assays that UBXN1 had a negative relationship with IFN-β, suggesting that TGEV infection promoted UBXN1 to inhibit the production of IFN-β in the late stage of infection to augment proliferation. The detailed mechanism of this process is worthy of further research.

In the present study, we identified 12 candidate proteins that potentially interact with TGEV-S1 in porcine small intestinal epithelial cells. Most of these proteins are involved in protein synthesis and transport, biological signal transduction, and negative regulation of signaling pathways; therefore, it is likely that TGEV-S1 has multiple functions in intracellular protein-related processes. We further confirmed the interaction between TGEV-S1 and UBXN1 and found that UBXN1 expression increased after TGEV infection. Moreover, UBXN1 negatively regulated TGEV-induced IFN production. We reported for the first time the interaction between TGEV-S1 and the UBXN1 protein in IPEC-J2 cells and found that UBXN1 plays an important role in the replication of TGEV in cells. In summary, the UBXN1-S1 complex may have a regulatory effect on TGEV infection-induced IFN production. These findings will lay a foundation for further understanding TGEV pathogenesis.
